# The Multifaceted Role of Rad9 in the DNA Damage Response of *Saccharomyces cerevisiae*

**DOI:** 10.1002/yea.70011

**Published:** 2026-03-04

**Authors:** A. Kiely, F. O'Halloran, P. Young, N. F. Lowndes, M. Grenon, K. Finn

**Affiliations:** ^1^ Department of Biological Sciences Munster Technological University Bishopstown Cork Ireland; ^2^ School of Biochemistry and Cell Biology University College Cork Cork Ireland; ^3^ School of Biological and Chemical Sciences, College of Science & Engineering University of Galway Galway Ireland; ^4^ Centre for Chromosome Biology, Biomedical Sciences Building, School of Biological and Chemical Sciences, College of Science & Engineering University of Galway Galway Ireland; ^5^ Department of Analytical, Biopharmaceutical and Medical Sciences, School of Life Sciences, Faculty of Science and Health Atlantic Technological University Galway Ireland

**Keywords:** cancer, DNA damage checkpoint, DNA damage response, DNA repair, double‐strand breaks, Rad9

## Abstract

To maintain the integrity of the genome, cells have evolved a complex signalling system, termed the DNA damage response (DDR), which detects DNA damage and promotes DNA repair. To date, over 600 proteins have been identified that play an integral role in the DDR. *RAD9*, encoding a DDR mediator protein, was the prototypical DNA damage checkpoint gene, establishing the genetic regulation of transient cell‐cycle delays upon DNA damage. Rad9, identified 38 years ago in the budding yeast *Saccharomyces cerevisiae* as a damage‐dependent cell‐cycle regulator, is now known to regulate additional responses to DNA damage including both cell‐cycle recovery and repair. The Rad9 protein is extensively phosphorylated both during a normal cell cycle and following DNA damage and several of these modifications have been linked to specific Rad9 roles within the DDR. Proteins structurally and functionally related to Rad9 exist in mammalian cells (e.g., 53BP1, BRCA1, MDC1) and insights into their regulation and mechanism of action have been informed by studies in yeast. This review will discuss the cellular mechanisms governing the DDR with an emphasis on the multifaceted role of Rad9 in sensing and responding to DNA damage, and how phosphorylation events regulate its function within the DDR. As the cellular events governing the DDR are well conserved, discoveries in yeast can be extrapolated to humans and may lead to the identification of additional novel protein targets, with several DDR inhibitors currently in clinical use or showing promise in clinical trials.

## Summary

- The DNA damage response is a conserved signalling cascade that arrests the cell cycle and promotes DNA repair.
- Rad9 functions as a key mediator to couple DNA damage sensing to checkpoint activation.
- Rad9 function is regulated by both cell cycle‐ and DNA damage‐dependent phosphorylation.
- Rad9 is recruited to sites of DNA damage by histone‐dependent and histone‐independent mechanisms.

## 1 Introduction

Cells are constantly exposed to DNA‐damaging agents arising from both endogenous and exogenous sources. Endogenous DNA damage can result from replication errors and by‐products of normal cellular metabolism, such as free radicals. Exogenous sources include radiation (e.g., ultraviolet [UV] and ionising radiation [IR] from solar or radon, respectively), and small molecules that directly cause double‐strand breaks (DSBs), termed clastogens, examples of which include alkylating agents, topoisomerase inhibitors, and chemical agents such as benzene (Huang and Zhou 2021). DSBs are the most detrimental form of DNA damage and can lead to gross chromosomal rearrangements (GCRs) that promote tumourigenesis (Hanahan 2022). DSBs arise from many sources including replication errors (e.g., fork collapse or passage over single‐strand breaks, SSBs, and single‐stranded DNA, ssDNA), site‐specific endonucleases, trapped topoisomerases, ionising radiation and multiple clastogens (e.g., arsenic, benzene, ethylene oxide, etc.) (Huang and Zhou 2021; Waterman et al. 2020).

**Figure 1:**
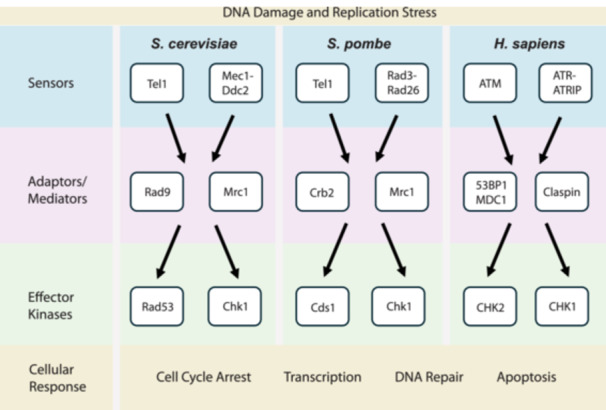
DNA damage response in yeast and humans. Simplified overview of the signal transduction network activated in response to DNA damage or replication stress. Checkpoint activation is initiated by sensor proteins, which are the apical kinases in the signal transduction pathway. Transduction of the checkpoint signal is mediated by adaptor proteins, which transduce the signal from the apical kinases to downstream effector kinases. The effector kinases phosphorylate downstream targets, which result in the various responses to DNA damage, including cell cycle arrest, activation of transcription, DNA repair and apoptosis.

To protect the integrity of the genome, cells have evolved a complex signalling system termed the DNA damage response (DDR), which detects DNA damage and promotes DNA repair (Figure 1). The first step is DNA damage sensing, which is achieved through a series of cell‐cycle stage‐specific mechanisms that detect genotoxic insults and trigger specific downstream cellular responses (Groelly et al. 2023; Pizzul et al. 2022; Waterman et al. 2020). In *S. cerevisiae*, DNA damage signalling, also called the DNA damage checkpoint, is initiated by two key sensor proteins belonging to the phosphatidylinositol 3‐kinase‐related kinase (PIKK) family, namely Tel1 and Mec1, which are specific to DSBs and ssDNA, respectively. Tel1 and Mec1 are the budding yeast homologues of the mammalian ATM and ATR kinases. In mammalian cells, a third PIKK, DNA‐PKcs also contributes to signalling DSBs but functions primarily in joining broken chromosome ends. Signal transduction is mediated in yeast by the Rad9 protein, with 53BP1/BRCA1/MDC1 adaptor proteins providing ‘mediator’ function in human cells. Mediators largely function to facilitate protein‐protein interactions, for example, bringing Tel1/ATM and Mec1/ATR into close proximity to the Rad53/CHK2 and Chk1/CHK1 effector kinases. This facilitates their activation, which in turn allows these effector kinases to phosphorylate their downstream targets, resulting in cell‐cycle arrest, activation of transcriptional programmes, regulation of DNA repair or, in human cells, if the damage is beyond repair, apoptosis or autophagy (Juretschke and Beli 2021; Lanz et al. 2019; Waterman et al. 2020). If the damage is successfully repaired, the checkpoint response must be deactivated to allow the cell cycle to resume in a process termed recovery. Alternatively, the checkpoint signal from persistent DNA damage can sometimes be overridden in a process termed adaptation, in which cells with unrepaired DNA damage re‐enter the cell cycle (Groelly et al. 2023; Pizzul et al. 2022; Waterman et al. 2020). Such adaptation to checkpoint signalling can result in increased mutagenic load, cellular senescence, apoptosis, inflammation, accelerated aging and carcinogenesis.

*RAD9* was the first DNA damage checkpoint gene identified in a pivotal study by Weinert and Hartwell (1988). Since then, pioneering studies have elucidated several important functions for Rad9 in the DDR. This review focusses upon the multifaceted role of Rad9 in sensing and responding to DNA damage, and how our understanding of its central role in the DDR has grown significantly since its discovery as a checkpoint gene over 38 years ago. In humans, defects in checkpoint proteins have been implicated in cancer and other genetic disorders including neurodegenerative diseases, immune deficiency disorders and developmental defects (Andarawi et al. 2025; Groelly et al. 2023). Of particular interest, cancer‐associated mutations have been identified in several DDR proteins, including the ATM and ATR apical kinases, and the BRCA1 adaptor protein; further understanding their biological effect may lead to the development of novel anti‐cancer therapeutics (Fu et al. 2022; Q. Li et al. 2023). The precedent for this is the well‐known use of poly (ADP‐ribose) polymerase (PARP) inhibitors to selectively kill tumours harbouring inactivating mutations in *BRCA1* (recently reviewed by Curtin and Szabo 2020; Q. Li et al. 2023). As such, elucidating the complexities of conserved DDR signalling networks can provide fundamental mechanistic insight into how DNA damage is detected and resolved, which in turn may open opportunities for new therapeutic interventions in disease. Initial discoveries made in model eukaryotic organisms, such as yeast, have been extended to humans and have provided the framework for the subsequent development of novel mechanism‐based therapeutics (recently reviewed by Groelly et al. 2023; Jordan et al. 2025; S. Lee et al. 2025).

## 2 DNA Damage Checkpoint Activation

The DNA damage checkpoint is orchestrated by proteins and protein complexes involved in sensing DNA lesions and transducing the DNA damage signal to downstream effector kinases, which in turn regulate downstream effector proteins. These signalling networks are structurally and functionally highly conserved between yeast and humans. The main *S. cerevisiae* proteins involved in the DNA damage checkpoint have structural and functional equivalents in *Schizosaccharomyces pombe* and humans (Table 1). While this review focuses on *S. cerevisiae*, and draws parallels with the human DDR, a comprehensive overview of the main *S. pombe* signalling kinases is presented by Cussiol et al. (2019).

**Table 1:** Early DNA damage checkpoint proteins and complexes in *Saccharomyces cerevisiae* and their *Schizosaccharomyces pombe* and structurally related human equivalents. This table summarises early DNA damage checkpoint proteins and protein complexes in *S. cerevisiae*, highlighting their structural and functional counterparts in *S. pombe* and humans. Both individual proteins and multi‐protein complexes are indicated to clarify their roles in checkpoint signalling.

Class of protein	*S. cerevisiae*	*S. pombe*	*H. sapiens*	Function
Sensors	Tel1	Tel1	ATM	PIKK recruited by MRX; sensing and transducing the checkpoint signal at minimally processed DSB ends.
	Mec1‐Ddc2	Rad3‐Rad26	ATR‐ATRIP	Principal PIKK is involved in sensing and transducing checkpoint signals. Binds RPA‐coated tracks of ssDNA.
	Ku70‐Ku80	Ku70‐Ku80	KU70‐KU80	Initial sensing of DSBs; binds and protects DNA ends. DNA end tethering. Important for DNA repair *via* NHEJ.
	Mre11‐Rad50‐Xrs2 (MRX)	Rad32‐Rad50‐Nbs1	MRE11‐RAD50‐NBS1 (MRN)	Multi‐functional complex with nuclease and ATPase activity involved in initial sensing and signalling of DSBs. DNA end tethering. Role in DSB repair *via* both HR and NHEJ.
	Ddc1‐Mec3‐Rad17	Rad9‐Hus1‐Rad1	RAD9‐HUS1‐RAD1	Heterotrimeric complex, structurally related to PCNA, also called the 9‐1‐1 complex. Sliding clamp involved in DNA damage signal transduction and recruitment/activation platform for other checkpoint proteins.
	Rad24‐Rfc2‐5	Rad17‐Rfc2‐5	RAD17‐RFC2‐5	Loading of 9‐1‐1 complex at sites of DNA damage.
	Dpb11	Cut5	TOPBP1	Replication initiation protein and checkpoint sensor recruited to sites of damage with functions in Mec1/ATR activation. Facilitates interaction between 9‐1‐1 complex and other DNA damage proteins at DNA lesions.
Replication‐associated factor	Dpb3‐Dpb4	Dpb3‐Dpb4	POLE4‐POLE3/CHRAC17	Sub‐complex of DNA polymerase ε. Role in replication and fork stability and promotion of replication checkpoint signalling. Facilitates chromatin reassembly and histone deposition at DSBs.
	Elg1	Elg1	ATAD5	Alternative RFC complex. Unloads PCNA from chromatin to maintain genome stability and facilitate the repair of DSBs.
DSB processing	Sae2	Ctp1	CtIP	Regulatory protein that functions with MRX/MRN complex for initial end resection of DSBs.
	Exo1	Exo1	EXO1	5′‐3′ exonuclease involved in recombination and DNA repair. Facilitates long‐range resection.
	Dna2	Dna2	DNA2	5′ endonuclease that functions with Sgs1 to resect DSBs. Involved in Mec1/ATR activation.
	Sgs1	Rqh1	BLM	RecQ‐like helicase that functions with Dna2 to process DSBs.
	Rfa1‐Rfa2‐Rfa3 (RPA)	Rad11‐Ssb2‐Ssb3	RPA1‐RPA2‐RPA3	Binds to ssDNA and protects against ssDNA degradation. Recruits DNA checkpoint/repair proteins, e.g. Mec1‐Ddc2/ATR‐ATRIP.
Adaptors/Mediators	Rad9	Crb2	53BP1; BRCA1; MDC1	DNA damage signal transduction and DSB repair. Required for Rad53 and Chk1 activation.
	Mrc1	Mrc1	Claspin	Involved in S‐phase checkpoint activation. Required for Rad53 activation in response to replication stress.
	Slx4	Slx4	SLX4	Recruits and coordinates structure‐specific nucleases at sites of damage. Role in DNA repair.
	Rtt107	Brc1	PTIP	Recruits repair factors to stalled replication forks or sites of DNA damage.
Methyl‐transferase	Dot1	Set9	DOT1L	Histone methyltransferase. Regulates chromatin structure and checkpoint signalling by facilitating recruitment of Rad9 to chromatin.
Effector kinases	Rad53	Cds1	CHK2	Effector kinase in the DNA damage checkpoint.
	Chk1	Chk1	CHK1	Effector kinase in the DNA damage checkpoint.
Effectors	Dun1	N/A	N/A	Acts downstream of Rad53 to regulate DNA damage and replication checkpoints. Promotes transcription of DNA damage‐inducible genes. No direct equivalent in fission yeast or human cells.
	Pds1	Cut2	Securin	Downstream target of Chk1. Prevents premature sister‐chromatid separation ensuring cells do not enter anaphase until damage is repaired.
Phosphatases	Ptc2, Ptc3	Ptc2, Ptc3	PPM1A, PPM1B, PPM1D	PP2C family phosphatases. Dephosphorylate checkpoint kinases to promote recovery.
	Pph3	Pph3	PPP4C	Catalytic subunit of PP4 complex. Involved in checkpoint recovery.
	Psy2	Psy2	PPP4R3	Regulatory subunit of PP4 complex. Modulates phosphatase activity.
	Tpd3	Paa1	PPP2R1A, PPP2R1B	PP2A scaffold subunit. Required for PP2A holoenzyme function during DNA damage checkpoint recovery.
	Cdc55	Pab1	PPP2R2A	PP2A regulatory B subunit. Negatively regulates DNA damage checkpoint signalling to promote checkpoint recovery.

A prerequisite for DNA damage checkpoint activation is sensing of the damaged DNA. DSBs are the most detrimental form of DNA damage and are initially and rapidly bound by both the Ku70‐Ku80/KU70‐KU80 and Mre11‐Rad50‐Xrs2/MRE11‐RAD50‐NBS1 (MRX/N) complexes. The Ku70‐Ku80 complex serves as a platform for the recruitment of several proteins involved in the non‐homologous end joining (NHEJ) repair pathway. This repair pathway functions in all cell‐cycle stages but is most important in the G1 phase when DNA end resection, and thus repair by homologous recombination (HR), is less active. Resection at DSB ends is regulated by phosphorylation events, specifically by cyclin‐dependent kinases (CDKs). CDKs, together with their cell‐cycle stage‐specific cyclin partners, regulate all major cell‐cycle events such as DNA replication and mitosis. While *S. cerevisiae* has one essential CDK, Cdc28, five CDKs (CDK1, 2, 3, 4, 6) regulate cell‐cycle events in humans (Pellarin et al. 2025). CDK activity plays a complex role in the DDR, regulating repair pathway choice and promoting specific protein recruitment to sites of damage (Kciuk et al. 2022).

In budding yeast, recruitment of Tel1 to sites of DNA damage is dependent on the MRX complex, where the Xrs2 subunit harbours a Tel1‐interacting domain, which mediates Tel1‐dependent checkpoint activation before DNA‐end processing (D'Amours and Jackson 2001; Grenon et al. 2001; Nakada et al. 2003; Usui et al. 2001). In S and G2 phases, high CDK activity promotes short‐range 5′–3′ resection of the DSB ends by the MRX complex and Sae2 (the yeast equivalent of CtIP), which displaces the Ku70‐Ku80 complex from DNA ends (Cannavo and Cejka 2014; Marini et al. 2019). The partially resected DNA ends are further resected by the Exo1/EXO1 nuclease and the nuclease‐helicase complex Dna2‐Sgs1/DNA2‐BLM.

The ssDNA revealed by resection is rapidly bound by replication protein A (RPA), which, in turn, serves as a platform to recruit the Mec1‐Ddc2/ATR‐ATRIP complex *via* Ddc2/ATRIP. Thus, these PIKK complexes ‘sense’ DSBs indirectly, requiring prior resection. Resection also triggers the switch from Tel1/ATM, which binds minimally processed DSBs *via* the MRX/MRN complex, to Mec1/ATR‐dependent checkpoint signalling (Cruz‐García et al. 2014; Haahr et al. 2016; Langerak et al. 2011; Mimitou and Symington 2008). Full activation of Mec1‐Ddc2/ATR‐ATRIP requires additional activator proteins that are independently recruited to the site of DNA damage; the Ddc1/RAD9 subunit of the 9‐1‐1 complex, the replication initiation factor Dpb11/TOPBP1, and the Dna2/DNA2 nuclease (Yates et al. 2025). First, the 9‐1‐1 checkpoint clamp, consisting of Ddc1‐Mec3‐Rad17/RAD9‐HUS1‐RAD1, is loaded onto DNA by the Rad24‐Rfc2‐5/RAD17‐RFC2‐5 clamp loader. The 9‐1‐1 complex assembles at the 5′ recessed end of the ds/ssDNA junction. In yeast, Mec1‐dependent phosphorylation of Ddc1 on T602 residue creates a docking site for the replication initiation factor Dpb11 (equivalent to human TOPBP1), which binds this residue *via* its C‐terminal tandem BRCT domains (Navadgi‐Patil and Burgers 2008, 2009; Pfander and Diffley 2011; Puddu et al. 2008). The mechanism of Mec1 activation is dependent on the cell‐cycle stage at which DNA damage occurs, with Ddc1 activating Mec1 in G1, S and G2/M phases, with Dpb11 playing a role in S and G2/M phases, and Dna2 further contributing to Mec1 activation in S phase (Kumar and Burgers 2013; Navadgi‐Patil et al. 2011; Navadgi‐Patil and Burgers 2009). Dpb11‐mediated Mec1 activation is regulated by CDK‐dependent phosphorylation, while Ddc1 can activate Mec1 independently of CDK activity, which reflects their differential control during the cell cycle.

To transduce the signal from the activated Tel1/ATM and Mec1/ATR kinases to the downstream effector kinases Rad53/CHK2 and Chk1/CHK1, the proteins must be brought into close proximity to one another on DNA. Interestingly, the first biochemical function described for Rad9 was consistent with such a molecular adaptor or scaffold function at sites of DNA damage where Mec1‐activated Rad9 catalyses the activation of Rad53 by *in trans* autophosphorylation (Gilbert et al. 2001; reviewed by Waterman et al. 2020). The specific mechanisms governing Rad9 recruitment and the functional protein domains/protein modifications associated with these interactions, in addition to subsequent activation of the downstream effector kinases, are discussed in detail below.

## 3 Structure and Function of the Rad9 Checkpoint Protein

**Figure 2:**
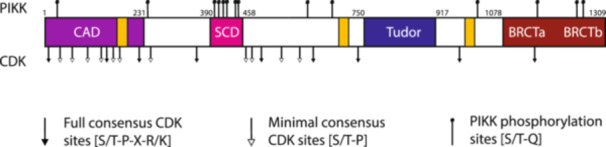
Domain structure of the Rad9 protein. The main structural features are indicated as coloured boxes, while the numbers indicate the amino acids corresponding to the domain boundaries. CAD: Chk1 activation domain (purple); NLS: nuclear localisation signal (orange); SCD: serine/threonine cluster domain (pink); Tudor: tandem Tudor domain (blue); BRCT: tandem BRCA1 carboxy terminus domains (brown). Also depicted are the 14 PIKK and 20 CDK putative phosphorylation sites.

The *RAD9* gene encodes a large 148 kDa protein containing 1309 amino acids (Toh and Lowndes 2003; Weinert and Hartwell 1990). To date, four functional regions have been characterised within the Rad9 protein; these comprise the Chk1 activation domain (CAD), SQ/TQ cluster domain (SCD), tandem Tudor domain, and tandem BRCA1 carboxyl terminal (BRCT) domain (Figure 2). No definitive Rad9 homologues have been identified in mammals. However, the 53BP1, BRCA1 and MDC1 proteins all share some structural, regulatory, and biochemical features with Rad9, with 53BP1 most closely resembling Rad9 (Pizzul et al. 2022; Raina et al. 2025; Ruff et al. 2020). Structurally, they all contain related tandem BRCT domains in their C‐terminus, which function to mediate protein–protein interactions, including oligomerisation of Rad9 itself, and often have a high affinity for phosphopeptide motifs (M. S. Lee et al. 2005; Lou et al. 2006; Soulier and Lowndes 1999; Stucki et al. 2005). Additionally, both Rad9 and 53BP1 contain a tandem Tudor domain upstream of the BRCT domains, which is required for their recruitment to damaged chromatin (Botuyan et al. 2006; Huyen et al. 2004).

The N‐terminus of Rad9, corresponding to the first 231 amino acids, comprises the CAD, which is required for the Mec1‐dependent phosphorylation and activation of the Chk1 effector kinase following genotoxic insult (Blankley and Lydall 2004). SCD, the second functional domain, is a cluster of 6 SQ/TQ phosphorylation sites spanning amino acids 390–458. Following DNA damage, these residues are phosphorylated in a Mec1‐ and Tel1‐dependent manner and promote the binding of the Rad53 effector kinase to Rad9 *via* the forkhead‐associated (FHA) domains of Rad53. In this capacity, Rad9 functions as a molecular adaptor‐scaffold, bringing Rad53 and Mec1 into close proximity and catalysing the Mec1‐dependent *in trans* autophosphorylation of Rad53 (Gilbert et al. 2001; Schwartz et al. 2002; Sun et al. 1998; Vialard 1998). This general adaptor‐scaffold function is also conserved in humans, where the putative Rad9‐related proteins facilitate the recruitment and accumulation of specific checkpoint and repair proteins at sites of damage, as well as promoting the ATM/ATR‐dependent phosphorylation of many downstream targets, including CHK1 and CHK2 (Raina et al. 2025; Ruff et al. 2020; Waterman et al. 2020).

Downstream of the SCD lies the tandem Tudor domain located between amino acids 750 and 917. The Tudor domain primarily functions to recruit Rad9 to the vicinity of DNA damage *via* binding of the Tudor domain to methylated lysine 79 of histone H3 (H3K79) and thus, initiating G1 and intra‐S phase checkpoint activation (Grenon et al. 2007; Huyen et al. 2004; Lancelot et al. 2007). Similar to Rad9, human 53BP1 contains a tandem Tudor domain, which is required for the recruitment of 53BP1 to sites of damage through its direct association with a distinct methylated histone, H4K20 (Botuyan et al. 2006). The fourth domain lies in the C‐terminus spanning amino acids 1078–1309 and contains tandem BRCT domains, which often recognise phosphorylated residues in target proteins and are present in many proteins involved in DNA damage checkpoint and repair pathways (Kruswick et al. 2024). The Rad9 tandem BRCT domains are required for all Rad9‐dependent functions in the DDR, and mediate Rad9 oligomerisation following DNA damage (Soulier and Lowndes 1999; Usui et al. 2009). The BRCT domains also interact with γ‐H2A, facilitating the recruitment of Rad9 to damaged chromatin (Hammet et al. 2007; Javaheri et al. 2006). Similarly, in humans, the Rad9 homologue MDC1 contains tandem BRCT domains in its C‐terminus, which bind γ‐H2AX after DNA damage, recruiting MDC1 to the chromatin flanking DNA damage (M. S. Lee et al. 2005; Stucki et al. 2005).

In addition to its Mec1/Tel1‐dependent hyperphosphorylation upon DNA damage, the Rad9 protein is also phosphorylated in a CDK‐dependent manner during a normal cell cycle (Abreu et al. 2013; di Cicco et al. 2017; Emili 1998; Gilbert et al. 2003; Pfander and Diffley 2011; Sweeney et al. 2005; Vialard 1998). This CDK‐dependent phosphorylation of Rad9 results in less mobility shift of the Rad9 protein in western blots and has been termed hypophosphorylation to distinguish these cell‐cycle‐dependent modifications from DNA damage‐dependent Rad9 hyperphosphorylation at PIKK sites. Rad9 contains 20 putative CDK sites ([S/T]‐P), 9 of which conform to the strict consensus sequence ([S/T]‐P‐X‐[R/K]) and 14 consensus sites ([S/T]‐Q) for phosphorylation by Mec1/Tel1 (Figure 2). To date, all 20 CDK sites and 9 PIKK sites have been shown to be phosphorylated in vivo (Chen et al. 2025). While several PIKK and CDK residues have been shown to be required for Rad9 interaction with other DDR proteins, including Mec1, Dbp11, Rad53 and Chk1, the biological function of many of these phosphorylation sites remains poorly understood.

## 4 Recruitment of Rad9 to DNA Damage Sites

In undamaged cells, Rad9 exists as a large soluble hypophosphorylated protein complex containing the heat shock protein (HSP) family chaperone proteins, Ssa1 and/or Ssa2, which promote the stability of the complex (Gilbert et al. 2001 and 2003). A small portion of this hypophosphorylated Rad9 complex is associated with chromatin during G1 and G2/M phases of the cell cycle in undamaged cells, which serves to enhance the speed and efficiency of the Rad9‐dependent response to DNA damage (Granata et al. 2010; Hammet et al. 2007). The primary role of Rad9 is to function as a molecular adaptor‐scaffold at sites of DNA damage to increase local effector kinase concentration and facilitate the Mec1/Tel1‐dependent phosphorylation of the downstream effector kinases Rad53 and Chk1 (Waterman et al. 2020). To facilitate checkpoint activation, additional large hypophosphorylated Rad9 complexes must first be recruited to DSBs. Recruitment can occur by histone‐dependent and histone‐independent mechanisms, the latter of which is cell‐cycle stage‐specific and dependent on CDK activity (Pizzul et al. 2022; Waterman et al. 2020).

**Figure 3:**
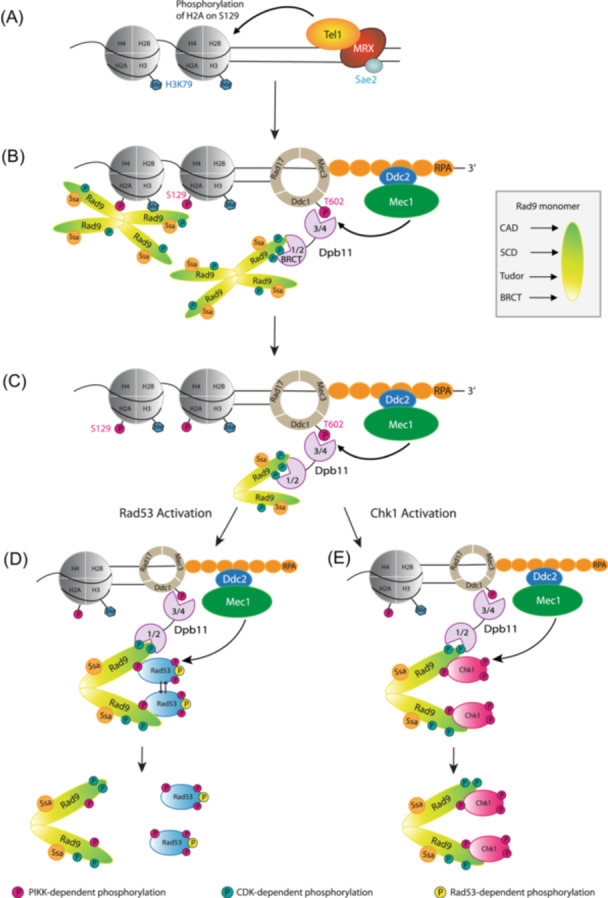
Model for Rad9 complex remodelling in response to DNA damage. (A) Following DSB formation, the Tel1 kinase is recruited to the DNA lesion by the MRX complex. Tel1 phosphorylates histone H2A on S129, and H3K79me is exposed at the site of DNA damage, possibly by the action of Dpb4‐Dpb3 (not shown). (B) In S and G2/M phases, high CDK activity promotes DNA end resection, which is initiated by the MRX complex and Sae2. The resultant ssDNA is coated by RPA and serves as a platform for Mec1‐Ddc2 recruitment and mediates the switch from Tel1‐ to Mec1‐dependent signalling. The 9‐1‐1 checkpoint clamp complex is loaded at the end of the ds/ssDNA junction. Mec1 phosphorylates Ddc1 on T602, which creates a docking site for Dpb11. In all cell‐cycle stages, Rad9 is recruited to DSBs by binding to γ‐H2A and methylated H3K79 via its BRCT and Tudor domains, respectively. The Rad9 monomer is shaded to represent the different domain boundaries. In S and G2/M phases, Rad9 can also be recruited by binding to Dpb11 via an interaction between the N‐terminal BRCT domain of Dpb11 and two phosphorylated CDK sites on Rad9. (C) Mec1‐dependent phosphorylation of Rad9 promotes remodelling of the large hypophosphorylated complex into a smaller hyperphosphorylated complex and provides docking sites for the Rad53 kinase. (D) Hyperphosphorylated Rad9 functions as an adaptor‐scaffold which catalyses Rad53 activation. Specifically, docking of multiple Rad53 molecules to the phosphorylated SCD (see Figure 2) results in their physical proximity, which promotes in trans autophosphorylation of Rad53 itself. Activated (hyperphosphorylated) Rad53 is predicted to have a lower affinity for hyperphosphorylated Rad9 and therefore dissociates from chromatin‐bound Rad9 to phosphorylate its own targets. (E) A small portion of Chk1 interacts with Rad9 in undamaged cells (not shown). Following DNA damage in G2/M, the Rad9‐Chk1 complex is recruited to sites of DNA damage facilitating the Mec1‐dependent phosphorylation of both Rad9 and Chk1. These phosphorylation events promote remodelling of Rad9, activation of Chk1, and subsequent release of the Rad9‐Chk1 complex from the DNA damage site.

Rad9 is recruited to chromatin *via* the histone‐dependent pathway, specifically by binding of its tandem BRCT domain to γ‐H2A (Hammet et al. 2007; Toh et al. 2006) and its Tudor domain to methylated H3K79 (Grenon et al. 2007; Wysocki et al. 2005). The DNA damage‐dependent γ‐H2A modification is formed by Mec1/Tel1‐dependent phosphorylation of serine residue 129 in the C‐terminus of histone H2A, equivalent to S139 of H2AX in mammalian cells (Downs et al. 2000; Rogakou et al. 1998; Figure 3A,B). Indeed, *h2a‐S129* mutants are defective in G1 checkpoint activation in response to DSBs due to impaired recruitment of Rad9 to sites of damage (Javaheri et al. 2006). Methylation of lysine 79 on histone 3 (H3K79me) is mediated by the histone methyltransferase Dot1/DOT1L and is constitutive and pervasive throughout the genome (Feng et al. 2002; Lacoste et al. 2002; Van Leeuwen et al. 2002). Interestingly, H3K79me is buried in the nucleosome core (Luger et al. 1997) and is inaccessible until DNA damage‐induced chromatin remodelling exposes the mark, thereby providing a docking site for Rad9 (Mohan et al. 2021; Wood et al. 2018). Recently, the Dpb4/POLE3/CHRAC17 histone fold protein interaction with Dpb3/POLE4 has been shown to facilitate this recruitment of Rad9 to DSBs (Casari et al. 2021). Genetic analyses suggest that Dpb4 functions in the same pathway as the Dot1 methyltransferase to promote Rad9 recruitment at DSBs. It is proposed that Dpb4‐Dpb3 would facilitate the re‐deposition of histones H3 and H4 at DSB ends, thus exposing methylated H3K39 as a docking site for Rad9.

An alternative Dpb11‐dependent pathway for Rad9 recruitment to DNA lesions also functions in G2/M phases of the cell cycle when CDK activity is high (Figure 3B). This histone‐independent pathway is mediated by the 9‐1‐1 checkpoint clamp complex and Dpb11, the yeast equivalent of TOPBP1 (Granata et al. 2010; Puddu et al. 2008). As mentioned previously, Mec1‐dependent phosphorylation of T602 of the Ddc1 subunit of the 9‐1‐1 checkpoint clamp complex promotes the interaction between Ddc1 and Dpb11. The C‐terminal tandem BRCT domains of Dpb11 bind to phosphorylated T602 on Ddc1 (Puddu et al. 2008), while the tandem N‐terminal BRCT domains of Dpb11 interact directly with Rad9 *via* two CDK‐phosphorylated residues on Rad9; S462 and T474 (Pfander and Diffley 2011). These interactions bring Rad9 into close proximity to Mec1 on damaged chromatin (Puddu et al. 2008). The Rad9‐Dpb11 interaction is cell‐cycle regulated, only occurring after Rad9 has been phosphorylated by CDK in G2/M phase (Pfander and Diffley 2011). The Dpb11–Rad9 interaction is also promoted indirectly by phosphorylation of a third CDK site (S11) on Rad9 (Granata et al. 2010). These interactions collectively function to recruit Rad9 to DNA lesions independently of histone marks.

Interestingly, studies using chromosomally integrated Lac operator arrays have shown that the Mec1‐dependent phosphorylation of Rad9 and Dpb11 at sites of DNA damage requires the unloading of chromatin‐bound DNA replication protein complex PCNA (proliferating cell nuclear antigen; Sau et al. 2019), a complex that plays an important role in DNA replication and repair (Sau and Kupiec 2020). The coordination of PCNA unloading with checkpoint activation requires a replication factor C‐like complex formed by Elg1/ATAD5 in association with Rfc2‐5. In *elg1∆* mutants, Dpb11 and Rad9 are recruited normally to sites of DNA damage during S phase, however they fail to be phosphorylated by Mec1, resulting in a lack of checkpoint activation (Sau et al. 2019). It is suggested that damage‐induced phosphorylation of Elg1 by Mec1 promotes unloading of PCNA from damaged chromatin, which is required for Mec1‐dependent phosphorylation of Dpb11 and Rad9 at sites of damage. Failure to unload PCNA from damaged chromatin may physically impede the interaction between Mec1 and Rad9/Dpb11, or its removal may generate a signal required for Mec1 to phosphorylate Rad9 and Dpb11 and activate the checkpoint response. In summary, Rad9 recruitment through the histone‐dependent pathway is essential in the G1 phase when CDK activity is low. As this pathway does not depend on cell‐cycle‐regulated protein–protein interactions, it ensures that a robust checkpoint response can occur in this phase of the cell cycle. The second histone‐independent pathway of Rad9 recruitment is dependent on CDK activity and ensures a robust checkpoint response in G2/M phase before entry into mitosis, a key control point consistent with *S. cerevisiae* cell‐cycle regulation.

## 5 Activation of the Downstream Effector Kinases

Recruitment of Rad9 to the chromatin flanking sites of DNA damage results in its hyperphosphorylation by Mec1/Tel1 (Figure 3C). PIKK‐dependent phosphorylation of the SCD domain of Rad9 promotes a conformational change in the large (> 850 kDa) hypophosphorylated Rad9 complex, remodelling it to a smaller (560 kDa) hyperphosphorylated complex (Van Den Bosch and Lowndes 2004) that allows Rad53 binding *via* its two FHA domains (Schwartz et al. 2002). Rad53 contains two FHA domains, FHA1 is located in the N‐terminal and FHA2 in the C‐terminal of the protein. The Rad9‐Rad53 interaction requires phosphorylation of multiple sites within the SCD to reach a required threshold for a stable Rad9‐Rad53 interaction (Schwartz et al. 2002). Both FHA domains of Rad53 bind phosphopeptides containing T390p, T398p, T410p, T427p, S435p and T457p of Rad9 in vitro, and it has been shown that full binding affinity between a single FHA domain and its target phosphopeptide involves a minimum of six amino acid residues encompassing the phosphorylation site (Durocher et al. 2000). As the six PIKK phosphorylation sites of the SCD sites are within a 69 amino acid motif, it is reasonable to suggest that multiple Rad53 FHA domains can bind a single Rad9 SCD motif thereby increasing their local concentration sufficiently to catalyse the *in trans* autophosphorylation required for Rad53 activation. Alternatively, a single Rad53 binds each SCD of two Rad9 molecules in the hyperphosphorylated Rad9 complex. This, too, would result in close‐proximity and Rad53 activation. It is tempting that the Ssa chaperone proteins may facilitate these inter‐ and intra‐molecular interactions. Once activated, phosphorylated Rad53 is predicted to have reduced affinity for the SCD and is released to phosphorylate its own targets. It is worth noting that the smaller hyperphosphorylated Rad9 complex also facilitates the Mec1‐dependent phosphorylation of Rad53 (Figure 3D), which has been suggested to promote Rad53 activation by *in trans* autophosphorylation (Gilbert et al. 2003; Sweeney et al. 2005; Usui et al. 2009; Van Den Bosch and Lowndes 2004).

In contrast to the Rad9‐Rad53 interaction, which requires both Rad53 FHA domains, only the Rad53 FHA1 domain is required for initiating an interaction between Rad53 and Ies4, a subunit of the INO80 chromatin remodelling complex (Kapoor et al. 2015). In the presence of DNA damage, the Ies4 subunit of the INO80 complex is phosphorylated by Mec1/Tel1, facilitating its direct binding to the Rad53 N‐terminal FHA domain and thus enhancing Rad53 activation (Kapoor et al. 2015). Interestingly, in vitro INO80 can activate Rad53 in the absence of Rad9, presumably by facilitating Rad53 *in trans* autophosphorylation. However, in vivo studies suggest that Rad9 and Ies4 function synergistically to phosphorylate Rad53 (Kapoor et al. 2015). Perhaps the physical proximity of both Rad9 and Ino80 in the chromatin flanking DNA damage together contributes to the full activation of Rad53. Once fully activated, Rad53 can also recruit additional Mec1 proteins to the vicinity of DNA damage thereby intensifying the checkpoint signal (Gilbert et al. 2001; Sweeney et al. 2005). A key downstream substrate of Rad53, once activated Rad53 has been released from the chromatin‐bound hyperphosphorylated Rad9 complex, is the Dun1 effector kinase. Dun1 promotes the transcriptional induction of several DNA damage‐inducible genes and genes encoding ribonucleotide reductase (RNR) subunits involved in the modulation of dNTP pools (De La Torre Ruiz and Lowndes 2000; Zhao and Rothstein 2002; Zhou and Elledge 1993). More recently, Dun1 has also been shown to play an important role in the stabilisation of the Pds1/Esp1 complex, which is required for G2/M arrest in response to chromosomal damage (Yam et al. 2020).

In contrast to Rad53 activation, a small portion of Rad9 and the Chk1 effector kinase interact constitutively in the absence of DNA damage (Abreu et al. 2013). CDK‐dependent phosphorylation of Rad9 during S, G2 and M phases of the cell cycle facilitates the Rad9‐Chk1 interaction independent of DNA damage, with phosphorylation of residues T143 and T125 promoting and inhibiting their interaction, respectively (Abreu et al. 2013). Following DNA damage, Rad9‐Chk1 is recruited to DSBs and phosphorylated by Mec1 (Figure 3E), leading to remodelling of the Rad9‐Chk1 complex (Gilbert et al. 2003; Tapia‐Alveal et al. 2009), Chk1 activation *in cis*, and the subsequent release of the smaller hyper‐phosphorylated Rad9‐Chk1 complex from DNA damage sites (Abreu et al. 2013; Y. Chen et al. 2009). Chk1 promotes Pds1/Securin stability *via* phosphorylation, thereby preventing separation of sister chromatids (Wang et al. 2001). As Pds1 stabilisation inhibits the metaphase‐anaphase transition, this checkpoint response functions to prevent anaphase entry.

In addition to its pivotal role in G1 and G2/M DNA damage checkpoints, Rad9 also cooperates with Mrc1/Claspin during S phase to control DNA replication initiation and elongation in response to DNA damage occurring during replication. The replication checkpoint is mediated by the adaptor protein, Mrc1 (Claspin in human cells), which facilitates the Mec1‐dependent phosphorylation of Rad53 in response to stalled replication forks during S phase (Berens and Toczyski 2012). However, in the presence of prolonged damage, stalled replication forks can collapse leading to DSBs, thus activating a Rad9‐dependent and DNA damage‐dependent checkpoint (Bacal et al. 2018; Serra‐Cardona et al. 2021). Recent studies have shown that Rad9 and Mrc1 cooperate both temporally and spatially to mediate the DDR in S phase. In MMS‐treated cells, repression of late‐firing replication origins is primarily Mrc1‐dependent, with Rad9 playing a role when MMS‐induced DNA lesions are bypassed, leading to Rad9 recruitment at DNA lesions left behind the replication fork. Mrc1 promotes a rapid, but transient, activation of Rad53 in early S phase to inhibit late‐origin firing under replication stress. As replication progresses and Mrc1‐associated forks decline, Rad9 sustains Rad53 activation through a signalling pathway requiring histone modifications and processing of ssDNA gaps generated after repriming past lesions. Taken together, Mrc1 provides an immediate response to replication stress, while Rad9 maintains checkpoint signalling to ensure ongoing forks remain stabilised and to delay mitosis until replication stress is fully resolved. Thus, the dual action of Mrc1 and Rad9 integrates early and late S‐phase responses to maintain genome integrity (Bacal et al. 2018; García‐Rodríguez et al. 2018; Moriel‐Carretero et al. 2019).

## 6 The Role of Rad9 in DSB Resection

**Figure 4:**
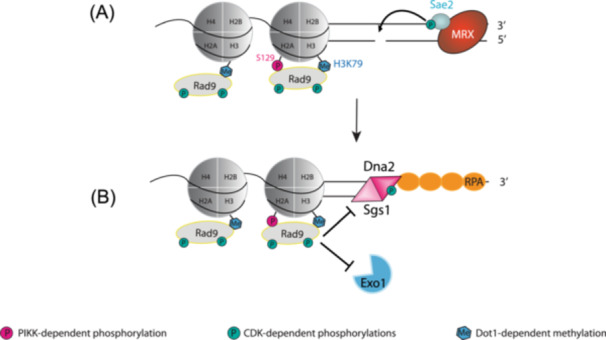
Role of Rad9 in double‐strand break resection. (A) In response to DSB formation (for simplicity, only one side of the DSB is shown), the MRX complex is recruited to DNA ends, where it promotes end tethering and initiates short‐range resection. Sae2, activated by CDK‐dependent phosphorylation, cooperates with the MRX complex to stimulate Mre11 endonuclease activity, cleaving the 5′ strand. This incision enables subsequent long‐range resection. Rad9 is recruited to chromatin surrounding the DSB as previously described. The Rad9 protein represented in the figure is likely to be an oligomer, but little is known about the specific structure of the Rad9 complex on chromatin at this stage of the process. (B) Long‐range resection is catalysed by the Exo1 nuclease and the Dna2‐Sgs1 nuclease‐helicase complex, with Dna2 activity enhanced by CDK phosphorylation. Rad9 limits extensive DNA end resection through checkpoint signalling and chromatin association, limiting long‐range resection by indirectly inhibiting the activity of Exo1 and physically inhibiting Sgs1 binding/persistence at DSB ends. The coordinated CDK‐dependent activation of Sae2 and Dna2, and the Rad9‐mediated control of long‐range resection, balances DNA end processing with checkpoint activation to ensure an appropriate DNA damage response.

Resection of DSB ends is a two‐step process comprising initial short‐range DNA end resection followed by more extensive long‐range resection. Short‐range resection is limited to the vicinity around the DSB and is mediated by the MRX complex and Sae2 (Figure 4A), while long‐range resection is catalysed by the Exo1 nuclease and the Dna2‐Sgs1 nuclease‐helicase complex (reviewed in Cejka and Symington 2021). Regulation of DSB end resection is a tightly controlled process that dictates DSB repair pathway choice. The canonical or classical non‐homologous end joining (cNHEJ) pathway requires no or very minimal resection to simply ‘end join’ DSBs. More extensive resection generates the long tracts of ssDNA required for HR, with the extent of resection required dependent upon the distance to the homologous partner (Waterman et al. 2020). In addition to components of ATP‐dependent nucleosome remodelling complexes (see below), DSB resection is controlled by CDK activity. Resection is promoted by the phosphorylation and activation of Sae2 (Huertas et al. 2008) and Dna2 (X. Chen et al. 2011) by CDK and is thus confined to the S/G2 phases of the cell cycle.

Rad9 limits excessive ssDNA formation during DSB resection by promoting Rad53 activation but also through Rad53‐independent mechanisms (Bonetti et al. 2015; Clerici et al. 2014; Dibitetto et al. 2015; Lazzaro et al. 2008; Ngo and Lydall 2015). Inhibition is dependent on the interaction of the Rad9 Tudor domain with methylated H3K79 and the 9‐1‐1 complex (Lazzaro et al. 2008; Ngo and Lydall 2015). The presence of Rad9 oligomers on the chromatin flanking DSBs limits long‐range resection by indirectly inhibiting the activity of Exo1 and physically inhibiting Sgs1 binding/persistence at DSB ends (Figure 4B; Bonetti et al. 2015). Cells lacking Rad9 exhibit faster 5′‐3′ resection mediated by both the Exo1 and Dna2‐Sgs1 pathways, and the Dna2‐Sgs1 pathway becomes essential in the absence of Sae2 (Ferrari et al. 2015). Indeed, it has been shown that Sae2 functions independently of the Mre11 nuclease to prevent Rad9 accumulation at DSBs (Colombo et al. 2019; Yu et al. 2018). The physical presence of Rad9 in the vicinity of DSBs limits the assembly of recombination factors at DSBs, which promotes DNA repair through HR sub‐pathways that require stable d‐loops such as long‐tract gene conversion, crossover recombination and break‐induced replication (Ferrari et al. 2020).

Interestingly, Fun30 (equivalent to human SMARCAD1), a component of a nucleosome remodelling complex of the Swr1‐like family, promotes DNA end resection by antagonising the inhibitory effect of Rad9; a role that is dependent upon its ATPase and helicase domains (X. Chen et al. 2012; Costelloe et al. 2012; Eapen et al. 2012). Following DNA damage, Fun30 is recruited to chromatin flanking the DSB, and promotes both Exo1‐ and Dna2‐Sgs1‐dependent end resection (X. Chen et al. 2012; Costelloe et al. 2012; Eapen et al. 2012). Increased accumulation of Rad9 at DSB ends has been observed in *fun30Δ* cells (X. Chen et al. 2012). Furthermore, resection proceeds faster in *fun30Δrad9Δ* cells compared with *fun30Δ* cells, and the requirement for Fun30 is less important in the absence of γ‐H2A or Dot1, both of which are required for recruitment of Rad9 to chromatin (X. Chen et al. 2012; Eapen et al. 2012). Recruitment of Fun30 to chromatin is cell cycle regulated and mediated *via* its interaction with the 9‐1‐1 complex and Dpb11 (Bantele et al. 2017). During late S to M phases of the cell cycle, CDK‐dependent phosphorylation of Fun30 promotes its recruitment to DSBs *via* interactions with the two N‐terminal BRCT domains of Dpb11. This mechanism is also conserved in human cells, where CDK‐dependent phosphorylation promotes the interaction between SMARCAD1 and TOPBP1 at sites of DNA damage (Bantele et al. 2017; Costelloe et al. 2012). As Rad9 also binds Dpb11 *via* the same interaction site on Dpb11 (Pfander and Diffley 2011), it has been suggested that Fun30 competes with Rad9 for Dpb11 binding at sites of damage. However, additional mechanisms, such as a role for Fun30 in the removal of Rad9 from chromatin or inhibition of Rad9 recruitment *via* nucleosome remodelling may also contribute but remain to be elucidated. Taken together, these studies suggest that chromatin‐bound Rad9 complexes function as a physical barrier to prevent the nucleolytic processing of DNA ends at sites of DNA damage.

## 7 DNA Damage Checkpoint Recovery

Once DNA damage has been repaired, the checkpoint must be deactivated so cells can resume cycling. Inactivation of checkpoint signalling, also called recovery, is achieved through the action of several protein phosphatases. To date, approximately 43 protein phosphatases have been identified in *S. cerevisiae*, with their roles often obscured by overlapping redundancies (Offley and Schmidt 2019). The most well‐characterised phosphatases belong to the phosphoprotein phosphatase (PPP), protein phosphatase metal‐dependent (PPM) and phospho‐tyrosine phosphatase (PTP) families.

A key dephosphorylation event for checkpoint deactivation is that of the Rad53 effector kinase (Pellicioli et al. 1999). It is dependent on phosphatases from the PPP (PP4 sub‐group) and PPM (protein phosphatase 2C sub‐group) families (Offley and Schmidt 2019; Ramos et al. 2019). The protein phosphatase 2C (PP2C) phosphatases Ptc2 and Ptc3 promote G2/M checkpoint recovery by binding to the Rad53 FHA1 domain and dephosphorylating Rad53, leading to Rad53 inactivation (Leroy et al. 2003). In contrast, the PP4 phosphatase complex, Pph3‐Psy2, has been shown to dephosphorylate Rad53 after DNA methylation damage in S phase (O'Neill et al. 2007; Travesa et al. 2008). Interestingly, PP4‐dependent Rad53 dephosphorylation also promotes DNA end resection by alleviating the inhibitory effect of Rad9 on the Dna2‐Sgs1 pathway (Villoria et al. 2019). It is suggested that down‐regulation of Rad53 activity by PP4 phosphatase activity controls resection at DSBs by preventing the recruitment of Rad9 to sites of DNA damage. As such, dephosphorylation of Rad53 by PP4 plays a dual role in both checkpoint recovery and in modulating end resection for DNA repair.

The PP2A phosphatase also plays an important role in downregulating the checkpoint response by preventing the Ddc1‐Dpb11 interaction at sites of DNA damage (Casari et al. 2023, 2025). The Cdc55 and Tpd3 subunits of the PP2A phosphatase attenuate DNA damage checkpoint signalling mediated by the 9‐1‐1 complex by binding of the Cdc55 subunit to Ddc1. This interaction prevents Dpb11 from binding to the Mec1‐phosphorylated T602 residue on Ddc1. Lack of Ddc1‐Dpb11 complex formation reduces Rad9 recruitment to sites of damage, thus preventing transmission of the checkpoint signal from Mec1 to Rad53 and preventing persistent cell‐cycle arrest (Casari et al. 2023, 2025).

A phosphatase‐independent mechanism for downregulating checkpoint signalling has also been described that requires the scaffold proteins Slx4/SLX4 and Rtt107/PTIP. The Slx4‐Rtt107 complex is recruited to sites of DNA damage *via* interactions between Slx4 and Dpb11 and between Rtt107 and damage‐induced γ‐H2A (Cussiol et al. 2015; Ohouo et al. 2010). Mec1‐dependent phosphorylation of Slx4 facilitates the interaction between Slx4 and the two N‐terminal BRCT domains of Dpb11 through the same domains involved in the Rad9‐Dpb11 interaction, thereby generating competition between Slx4 and Rad9 for Dpb11 binding (Ohouo et al. 2010, 2013). It has been proposed that the Slx4‐Rtt107 complex displaces Rad9 from the site of DNA damage, thereby counteracting the Rad9‐dependent activation of Rad53 (Cussiol et al. 2015; Dibitetto et al. 2015; Ohouo et al. 2013). A similar mechanism has been reported in human cells, where TOPBP1 mediates DNA repair control *via* 53BP1 and BRCA1 (Y. Liu et al. 2017). Together, these mechanisms ensure timely checkpoint inactivation and restoration of cell cycle progression once DNA repair is complete.

## 8 Future Perspectives

Despite extensive progress, the *S. cerevisiae* phosphoproteome continues to reveal additional levels of complexity in the regulation of the DDR. Thirty‐eight years after its discovery, Rad9 remains a key paradigm for understanding how multisite phosphorylation orchestrates checkpoint activation, signal amplification and recovery. Several important phosphorylation sites have been identified and shown to play important roles in Rad9 function, however the role of others, of which there are many, remains to be elucidated. Defining the specific roles of individual phosphorylation events and the temporal coordination between kinase‐ and phosphatase‐mediated modifications represents an important area for further investigation. In particular, elucidating how specific phosphatases act on Rad9 both during normal cell‐cycle progression and following DNA damage will provide mechanistic insight into the pathways that regulate checkpoint proteins and allow the resumption of coordinated cell‐cycle progression once DNA damage has been repaired. As these mechanisms are often highly conserved between yeast and humans, dissecting the complex phosphorylation/dephosphorylation dynamics should inform understanding of analogous control mechanisms in human DDR mediators, for example, 53BP1, MDC1 and BRCA1. Integrating systematic phosphoproteomic analyses with mechanistic studies both in yeast and mammalian systems will therefore be essential to define how reversible phosphorylation governs genome stability across eukaryotes.

## Author Contributions

**A. Kiely, F. O'Halloran, P. Young, N. F. Lowndes, M. Grenon and K. Finn:** conceptualisation. **M. Grenon and K. Finn:** revised conceptualisation for journal/special issue and associated manuscript revisions. **A. Kiely, F. O'Halloran and K. Finn:** literature search and data curation. **A. Kiely:** writing – original draft. **A. Kiely, F. O'Halloran, P. Young, N. F. Lowndes, M. Grenon and K. Finn:** writing – review and editing. **F. O'Halloran and K. Finn:** supervision.

## Conflicts of Interest

The authors declare no conflicts of interest.

## Data Availability

Data sharing is not applicable to this article, as no data sets were generated or analysed during the current study.
